# Food Safety Knowledge and Practices among Saudi Mothers

**DOI:** 10.3390/foods7120193

**Published:** 2018-11-25

**Authors:** Wafa O. Ayaz, Anushree Priyadarshini, Amit K. Jaiswal

**Affiliations:** 1 School of Food Science and Environmental Health, College of Sciences and Health, Dublin Institute of Technology, Cathal Brugha Street, Dublin D01 HV58, Ireland; d15125240@mydit.ie (W.O.A.); amit.jaiswal@dit.ie or akjaiswal@outlook.com (A.K.J.); 2 School of Accounting and Finance, College of Business, Dublin Institute of Technology, Aungier Street, Dublin D02 HW71, Ireland

**Keywords:** food safety, food knowledge, food practices, food hygiene, foodborne illnesses, mothers

## Abstract

This study examines food safety knowledge and practices of mothers in Saudi Arabia. A total of 979 respondents participated in the study and completed a questionnaire that assessed their knowledge of food storage, food handling, usage, and maintenance of kitchen facilities, personal hygiene, and food poisoning. Results showed that mothers in Saudi Arabia had moderate knowledge of food storage (passing rate 64.9%) and usage and maintenance of kitchen facilities (passing rate 66.5%). While they had good knowledge of personal hygiene (passing rate 83.8%) and food poisoning (passing rate 78.5%), their knowledge with regard to food handling was poor (passing rate 30.4%). Results also highlighted that food safety knowledge and practices amongst mothers in Saudi Arabia improved with the level of education, while their age, employment status, monthly income, and number of children had no significant association with their food safety knowledge and practices. This research revealed the importance of education and that advance education and training program can further improve mothers’ food safety knowledge and practices and thereby result in reducing the risks of foodborne illnesses at homes.

## 1. Introduction

Food borne illnesses are a widespread public health concern and a burden on a nation’s economy [1,2]. World Health Organization (WHO) recognized foodborne illnesses and occurrences as a foremost public health threat globally of the 21st Century [3]. While everyone is vulnerable to foodborne illness, certain sections are regarded as more susceptible and thus more likely to suffer foodborne illnesses, hospitalizations, and death. These highest risk individuals, popularly known as the YOPI group include young, old, pregnant, and immunosuppressed [4,5]. Children due to their immature immune system and lower body weight are at a higher risk than adults for foodborne illnesses and these are described as one of the main factor for morbidity and mortality in developing countries [6]. About 2.2 million deaths each year in the developing world occur because of foodborne illnesses, an estimated 1.9 million of which are children. These are primarily caused by contamination of food and drinking water caused from poor sanitation or preparation [7].

Studies highlight that unsafe domestic food safety practices are increasingly linked with foodborne illnesses [8,9,10]. Moreover, as domestic kitchens are often used as a multipurpose area the risk of food contamination and spread of foodborne illnesses increases [11,12,13]. Domestic food handlers often cause contamination if they lack hygienic food handling practices [14]. Da Cunha et al. [15] also suggest that contamination can occur if foods are stored at the wrong temperature or inadequately packaged. While public awareness and concern about food related risks and diseases is increasing, the escalation in the number of foodborne illnesses indicates that domestic food handlers still lack adequate food safety knowledge leading to wrong food handling practices [16,17]. Scott [18] suggests that a considerable number of food borne illnesses are generally associated with practices in the domestic kitchens. Research highlights that about 50 and 87% of reported foodborne disease incidents arises within the home [19,20,21,22], enhanced by a lack of education and awareness about food safety and food handling practices [23]. 

Recognizing that knowledge is essential to safe food handling, many studies have focused on improving the food safety education of consumers [24,25,26]. Haapala and Probart [27] reported that in developed countries national initiatives are launched to find techniques to effectively educate food consumers. In developing countries, however, Ministry of Health in Saudi Arabia pointed out that there have been increasing number of foodborne illnesses in the country [28], 255 incidences occurred in 2011 alone resulting in 2066 people falling ill, majority of whom were children [29]. Nonetheless, [16] highlight that only limited research has focused on obtaining information on food safety knowledge and practices associated with improper food handling at home in Saudi Arabia. Consequently, inadequate efforts have been undertaken to reduce the risks by development of effective health education programs. As mothers are primarily the food handlers at home; to safeguard children’s health and wellbeing assessing mother’s beliefs and behavior and gathering information on how food becomes unsafe in the home is essential in order to reduce food hazards [30]. Also, as food preparation, handling, and storage at home cannot be regulated, for maintaining food safety at the vulnerable end of the food chain it is vital to educate domestic food handlers about the potential risks of foodborne diseases and guide safe food handling practices at home. This study therefore aims to assess food safety knowledge and practices among mothers in Saudi Arabia, so that effective health education programs can be developed with the help of sufficient information on the knowledge and practices of the target groups.

## 2. Materials and Methods 

### 2.1. Questionnaire Design

To survey mothers in Saudi Arabia for their food safety knowledge and food handling practices a closed-ended questionnaire with multiple-choice questions was designed. The questionnaire development was guided by validated questionnaire used in similar studies [31,32,33,34]. Appropriate modifications were made to the questionnaire to fit the popular habits and traditions of domestic food handlers in Saudi Arabia. 

The questionnaire consisted of 32 multiple-choice questions, categorized into six sections. Demographic information of each participant, such as age, educational level, employment status, income level, and number of children was asked in the first section. The questionnaire then seeks information about the participant’s knowledge of food safety and practices in home kitchens through five sections, namely: knowledge of food storage (information collected through five questions), knowledge of food handling (via four questions), knowledge of the usage and maintenance of kitchen facilities (through 6 questions), knowledge of personal hygiene (through six questions) and knowledge of food poisoning (via six questions). For validation of the questionnaire a pilot study was conducted amongst food safety and business management professionals. The questionnaire was later distributed both manually and electronically to mothers in Saudi Arabia.

### 2.2. Data Collection

Surveys are an efficient tool to collect information from large volume of respondents in a short span of time [35]. The participants for this survey were selected from across Saudi Arabia for maximum coverage. However, the sampling frame covered the various demographic aspects of the questionnaire like age, place of residents, and educational level so as to safeguard a non-bias sampling. The questionnaires were distributed both manually and electronically and response collected between September and December 2017. A total of 1000 Saudi mothers were contacted to participate in the study, however, 21 surveys were removed as they had more than one option selected or the participants did not complete the questionnaire as they did not want to respond to the questionnaire erroneously. Thus, 979 surveys were included in this research.

### 2.3. Data Analysis

Statistical Package for the Social Sciences (SPSS) (IBM Corporation, Armonk, NY, USA) version 24 statistical package was used to analyze the data. For each question the participants were awarded one point when they answered correctly and a zero for incorrect answers and their percentages calculated for each section namely knowledge food storage, knowledge of food handling, knowledge on usage and maintenance of kitchen facilities, knowledge on personal hygiene and knowledge of food poisoning. Mean score and standard deviation for each section was analyzed, and pass rates calculated with participants who answered more than half of the questions in the section correctly attaining a pass. If the participants achieved a passing rate of 60.0% or above, they were considered to have a high level of knowledge, while a pass rate of less than 30.0% was regarded as a poor level of knowledge, and pass rates between 30–60% were considered as moderate level of knowledge. Furthermore, the relationship between the demographic characteristics and knowledge of food safety in homes was also analyzed.

Non-parametric analysis is the most appropriate method for analyzing the data when data follows a skewed distribution [36]. Therefore, for analyzing this data, non-parametric tests (Chi-square (×2) and Kruskal-Wallis) were used. Chi-square (×2) test was used to compare the different demographics for their food safety knowledge and practices while Kruskal-Wallis test was used in the case of demographics of three or more independent samples, i.e., age, education level, employment status and number of children in our survey.

## 3. Results and Discussion

### 3.1. Samples Profile

Table 1 presents the demographic characteristics of the sample. 40.7% of the respondents were between the age of 36 and 50, with majority (51.5%) being housewives. The highest educational level of the respondents (76%) was University Degree and most respondents (44%) had four or more children.

### 3.2. Food Safety Knowledge and Practices of Saudi Mothers

#### 3.2.1. Knowledge on Food Storage

Table 2 presents a summary of the knowledge of domestic food handlers about food storage. Results show that majority of the respondents (73.6%) were aware that frozen foods must be purchased at the end of the shopping. Other similar studies highlight that in Lebanon 59.7% [32], in Greece 55.3% [37], in Jordan 73.6% [34], participants are aware of the correct time of purchasing frozen foods when shopping. However, only 30.2% respondents knew the correct temperature for storing frozen food. Farahat et al. [16] also highlight the poor knowledge of food safety in women in Saudi Arabia during their food purchasing and storage. A total of 70% of the respondents were knowledgeable about not freezing thawed meat for later use and storing chunks of raw meat by slicing them into smaller pieces, sealing and storing them in the freezer (77.2%), and 37.8% respondents knew that for consuming freshly prepared food 3 h later, it must be put in the fridge, and reheated when ready to eat, however, almost equal number of respondents (33.1%) incorrectly thought that it can be covered and put on the cabinet.

The overall passing rate for this section was at 64.9%, indicating moderate knowledge of food storage. The results highlight that while mothers in Saudi Arabia have generalized knowledge about food storage they lack a detailed understanding which impacts their practices. Langiano et al. [38] also highlight that poor food storage practices greatly increase the risk of poor food safety practices and consequently of food borne diseases. Similar results were reported by Angelillo et al. [39] who explored the knowledge, attitudes and related behavior on foodborne diseases and food-handling practices among mothers of children attending public schools. 

#### 3.2.2. Knowledge on Food Handling

Table 3 summarizes the knowledge of food handling of Saudi mothers. 54.6% of respondents knew that fruits and vegetables should be washed in running cold water. Similar studies conducted in Canada highlight that 92.5% participants are aware that fresh produce must be washed with cold running water [40], 82% in South Africa [41], 51.4% in Lebanon [32], 72.8% in Greece [37], and 28.4% in Jordan knew the correct answer [34]. Respondents also had poor knowledge about the handling leftovers, with only 31.6% being aware of the correct way of heating leftovers and only 8.8% knowing when leftovers should be discarded. Additionally, most had poor knowledge about how raw meat should be thawed, the results were in line with findings from other similar studies like in China only 38.2% respondents knew the correct way to defrost food [31] and 58.3% in Brazil knew the correct answer [42]. Highlighting the importance of educating domestic food handlers so as to avoid cross-contamination and eliminate bacterial growth, to ultimately avoid foodborne illnesses [10,38]. 

The overall passing rate for this section was at 30.4%, highlighting poor knowledge of mothers in Saudi Arabia with regard to food handling. Farahat et al. [16] also highlight the poor knowledge of food handling in women in Saudi Arabia, while El-Sheikha [43] indicate that unsafe food handling increasingly results in bacterial food poisoning cases in Saudi Arabia. Trepka et al. [2] in their study of pregnant women and mothers in Miami, Florida also reported that the respondents had problematic food handling practices. Similar results have been reported by Parvathy et al. [44] when exploring food borne illnesses arising from home kitchens in Tamil Nadu, India. Angelillo et al. [39] also reported that mothers in Italy had poor knowledge about thawing food and handling leftovers. 

#### 3.2.3. Knowledge on Usage and Maintenance of Kitchen Facilities

Results of the knowledge of Saudi mothers on the usage and maintenance of kitchen facilities are presented in Table 4. Most respondents (76.3%) were aware that dishes must be cleaned immediately after meals and that the same chopping board used to cut meat should not be used to cut fruits (78.5% respondents). However, fewer number of respondents (42.3%) knew that the correct way to clean kitchen countertop is with detergent and warm water. Similarly, respondents had poor knowledge about the recommended temperature for fridges, and where in the fridge to place raw meat, with only 34.5% and 23% respectively knowing the correct answer. While in Portugal 69.5% of respondents knew the correct fridge temperature [45], and in Wales 84.0% of the elderly had awareness about the proper temperature of the refrigerator [46].

The overall passing rate for this section was at 66.5%, indicating moderate knowledge of usage and maintenance of kitchen facilities amongst mothers in Saudi Arabia. To avoid pathogenic growth or cross-contamination to maintain food safety it is critically important to have knowledge in usage and maintenance of kitchen facilities [38,46]. Similar results were observed by Usfar et al. [47] in their study of urban mothers in Indonesia. However, unlike the current study, Angelillo et al. [39] have reported a higher number of respondents being aware of correct way to clean kitchen countertop when exploring the knowledge of mothers of children attending public schools in Italy. 

#### 3.2.4. Knowledge on Personal Hygiene

Table 5 presents a summary of the knowledge of personal hygiene in Saudi mothers. Results show 65.3% respondents knew the correct way of washing hands was with running warm water using soap and then wiping them dry. Similarly, 60.5% knew that as long as gloves are worn it is safe to handle food with wound on the back of the hand. Mothers in Saudi Arabia had better knowledge in this regard as compared to results observed similar studies in China, with only 25.5% participants being aware of the correct answer [31], 29.1% in Brazil [42], 19.6% in Greece [37], and 23.0% in Jordan [34]. Almost all the respondents (95.8%) knew the correct way to wash hands after handling raw meat while only a fewer number (49.2%) were aware that people with diarrhea, fever, sore throat or flu symptoms should not cook for others. Similarly, 97.0% in South Africa [41], 75.6% in Ghana [48], 57.5% in Greece [37], and 96.1% of university students in Saudi Arabia indicated that they wash their hands before handling or preparing foods [49]. Results also indicate that an almost similar number of respondents (49.2% and 44.4%, respectively) regarded diarrhea, fever, sore throat, flu, and AIDS patients as not suitable to cook for others lest they can cause food contamination. This highlights the need and importance of capturing information on the knowledge and practices of the target groups and utilizing it for the development of effective health education programs. The overall passing rate for this section was at 83.8%, highlighting that mothers in Saudi Arabia had good knowledge with regard to personal hygiene. Fawzi and Shama [19] also reported that women working in Alexandria University, Egypt were very knowledgeable with regard to personal hygiene. Others studies have also suggested that food handlers have a significantly good knowledge of personal hygiene [50,51].

#### 3.2.5. Knowledge on Food Poisoning

Table 6 presents the knowledge of Saudi mothers on food poisoning. A total of 61.2% respondents knew that the most important method for preventing food poisoning was to keep it refrigerated until it’s time to serve it. Respondents had poor knowledge about which foods can cause food poisoning, with only 10.5% reporting that it can be caused by raw or undercooked beef and eggs. Other studies highlight that in China undercooked beef was regarded as a cause of food poisoning by 25.3% respondents, raw eggs by 8.9%, and both by 12.2% [31], in Portugal respondents were divided for the same as 12.5%, 19.0% and 43.8%, respectively [45], in South Africa 64.3% regarded raw eggs as a cause of food poisoning [41], 44.1% in Ghana [48], and 52.9% in Jordan considered undercooked eggs as unsafe [34]. While 45.4% respondents knew that *Salmonella* sp. poisoning can be prevented by fully heating food, most respondents (49.9%) did not know if bacteria could be killed by freezing at −18 °C but 53.2% were aware that raw beef or chicken are most likely to be contaminated with *Escherichia Coli (E. coli)*. 

The overall passing rate for this section was at 78.5%, indicating good knowledge of food poisoning amongst Saudi mothers. Lum et al. [50] also report that food handlers are knowledgeable with regard to food poisoning; Ahmed [14], however, reported that in Yemen mothers’ knowledge about food poisoning was not satisfactory. 

### 3.3. Association between the Demographic Characteristics of Participants and Their Food Safety Knowledge and Practices

**Figure 1:**
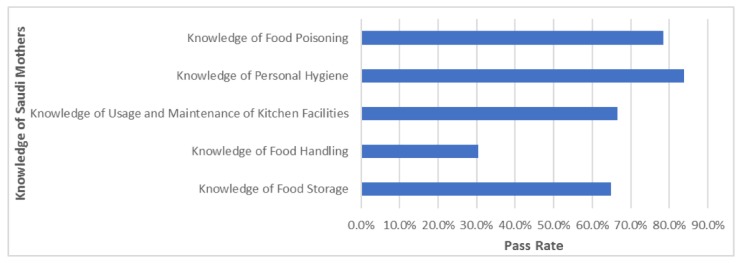
Pass rate for knowledge of food safety among Saudi mothers (Knowledge levels—Low: Pass rate up to 30%; Moderate: Pass rate 30–60%; High: Pass rate above 60%).

Table 7 presents the relationship between the demographic characteristics of Saudi mothers and their food safety knowledge and practices. Their mean score and overall passing rate using the non-parametric tests (Chi-square (×2) and Kruskal-Wallis) are also reported. The study highlights that while mothers in Saudi Arabia had good food safety knowledge, their food handling practices were poor, and there is scope for improvements in their food safety knowledge and practices (Figure 1). Results show that education is a significant factor that impacts the food safety knowledge and practices among Saudi mothers. On the other hand, age (N = 979; *p* value 0.330), number of children (N = 979; *p* value 0.181), employment status (N = 979; *p* value 0.891), and monthly income (N = 979; *p* value 0.356), do not have any significance with regard to impacting their food safety knowledge and practices. 

Mothers between the age of 36–50 year were better with food safety knowledge and practices with a pass rate of 41.6%. Housewives were more knowledgeable with a pass rate of 53.8% and those who had four or more children reported a better pass rate of 45.7%. Additionally, results showed that higher monthly income Saudi mothers were better with their food safety knowledge and practices and had a pass rate of 57.3%. 

The results correlate with other studies that highlight the importance of education and that of educational programs for mothers in improving their food safety knowledge and practices [39,47]. Furthermore, the findings highlight that the necessity of gathering information of domestic food handlers’ beliefs and behavior in order to reduce the risks of foodborne diseases and guide safe food handling practices at home. WHO [1] also suggest the advance education is comprehensively required so as to assist lessening foodborne illnesses and for comprehending the issue of why food safety is a globally epidemic. 

## 4. Conclusions

This study assessed the food safety knowledge and practices among mothers in Saudi Arabia. The information could be used as a starting point to design education and training programs that can further improve their knowledge and can be translated into better and safer food handling practices. Food safety efforts generally tend to focus on food supply chains and the household domain of food handling and practices gets limited attention. Food handlers in households need effective and methodical education and training to safeguard themselves and their families from getting food-borne illnesses. It is, therefore, recommended that authorities, researchers, educators, media, and food safety communicators should initiate education programs, with special focus on the high-risk groups like mothers and food handlers at home to advance the food safety knowledge and safer food practices. 

## Figures and Tables

**Table 1:** The demographic characteristics of mothers in Saudi Arabia.

Demographic Characteristics	N	Category	Respondents (n.)	Percentage (%)
Age	979	18–25	55	5.6
		26–35	358	36.6
		36–50	398	40.7
		51 and above	168	17.2
Children	979	1 child	186	19
		2 children	178	18.2
		3 children	184	18.8
		4 children or more	431	44
Educational level	979	Primary Education	22	2.2
		Secondary Education	213	21.8
		University Degree	744	76
Employment status	979	Housewife	504	51.5
		Employee	321	32.8
		Retired	95	9.7
		Other	59	6
Monthly income	979	2000–4999 SR *	117	12
		5000–6999 SR	110	11.2
		7000–9999 SR	206	21
		10,000 SR or more	546	55.8

* SR: Saudi Riyal.

**Table 2:** Respondents’ knowledge on food storage, pass rate, mean score and standard devastation.

Questions	N	Category	Respondents (n.)	Percentage (%)
1. When is the best time to purchase frozen food when shopping?	979	At the beginning of the shopping time	39	4
		**At the end of the shopping time**	721	73.6
		Whenever, does not matter	184	18.8
		Don’t Know	35	3.6
2. What is the optimal temperature for storing frozen food?	979	4 degrees Celsius	93	9.5
		0 degrees Celsius	209	21.3
		**−18 degrees Celsius or below**	296	30.2
		Don’t Know	381	38.9
3. What should be done with freshly prepared food that will be consumed 3 h later?	979	**Put in the fridge, then reheat when ready to eat**	370	37.8
		Put in the cupboard and reheat when ready to eat	271	27.7
		Put in the microwave oven	14	1.4
		Cover it and put it on the cabinet	324	33.1
4. How should chunks of raw meat be stored?	979	Store it directly in the freezer	90	9.2
		Slice it into smaller pieces, then store them in the freezer	119	12.2
		**Slice into smaller pieces, seal and store them in the freezer**	756	77.2
		Store it in a cool place	14	1.4
5. Should defrosted meat be frozen for later use?	979	Yes	124	12.7
		**No**	685	70
		Maybe	149	15.2
		Don’t Know	21	2.1
Pass Rate				64.9%
Mean ± standard deviation				2.8 ± 1.1

**Table 3:** Respondents’ knowledge on food handling, pass rate, mean score and standard devastation.

Questions	N	Category	Respondents (n.)	Percentage (%)
1. How should vegetables and fruits be washed?	979	Soak in Detergent	37	3.8
		Wash with hot water	41	4.2
		**Wash with running cold water**	535	54.6
		Soak in cold water, then wash	366	37.4
2. Of the following, which is the least safe way to defrost raw meat?	979	In the fridge	91	9.3
		**On Chopping Board**	176	18
		In Microwave oven	459	46.9
		In cold water in sealed package	253	25.8
3. Of the following, which is the correct way to heat leftovers?	979	Heat it to the temperature you prefer	569	58.1
		Reheat is not necessary if it’s during the summer	36	3.7
		**Heat until they are boiling**	309	31.6
		Do not know	65	6.6
4. What should be done if the leftovers are still not eaten completely?	979	**Discard them immediately**	86	8.8
		Put in the refrigerator immediately and reheat before consuming	741	75.7
		Store in kitchen and reheat before consuming	40	4.1
		As long as they smell good, eat them	112	11.4
Pass Rate				30.4%
Mean ± standard deviation				1.1 ± 0.8

**Table 4:** Respondents knowledge on usage and maintenance of kitchen facilities, pass rate, mean score, and standard devastation.

Questions	N	Category	Respondents (n.)	Percentage (%)
1. A fridge has three shelves; on which shelf do you think raw meat should be placed?	979	Top shelf	546	55.8
		Middle shelf	83	8.5
		**Bottom shelf**	225	23
		Does not matter	125	12.8
2. What is the recommended temperature for fridges?	979	12 °C	197	20.1
		**4 °C**	338	34.5
		0 °C	42	4.3
		Do not know	402	41.1
3. How long should leftovers be kept in the fridge?	979	**No more than 2 days**	494	50.5
		No more than 5 days	220	22.5
		As long as the food has not gone bad	217	22.2
		Do not know	48	4.9
4. Of the following, which is the correct way to clean the kitchen countertop and stove?	979	Clean with dry rag	7	0.7
		Clean with wet rag	36	3.7
		**Clean with detergent and warm water**	414	42.3
		All of the above	522	53.3
5. Of the following, which do you think is the correct way to wash dishes?	979	Soak in water, after several hours, wash with the same water	16	1.6
		**Wash immediately after meal**	747	76.3
		Wash in water basin, dry with dish cloth	162	16.5
		Other	54	5.5
6. Cutting meat on a chopping board and using the same chopping board for cutting fruit. Of the following, which are the correct ways?	979	Rinse the chopping board with hot water before cutting fruit	20	2
		Use the other side of the chopping board to cut fruit	79	8.1
		Clean the chopping board with detergent and hot water before cutting fruit	111	11.3
		**Use another chopping board to cut fruit**	769	78.5
Pass Rate				66.5%
Mean ± standard deviation				3.1 ± 1.2

**Table 5:** Respondents knowledge on personal hygiene, pass rate, mean score, and standard devastation.

Questions	N	Category	Respondents (n.)	Percentage (%)
1. Is it safe to handle food if you have a wound on the back of your hand?	979	Yes, as long as the wound is not infected	33	3.4
		Yes, as long as the wound has a bandage on it	274	28
		**Yes, as long as gloves are worn**	592	60.5
		Not at all	80	8.2
2. Of the following, which is the correct way to wash hands?	979	Wash with running cold water, wipe dry	70	7.2
		Wash with running warm water, wipe dry	69	7
		Wash hands with cold water in a basin, use soap and then wash hands with cold water in the basin, wipe dry	201	20.5
		**Wash hands with running warm water, use soap and then wash with running warm water, wipe dry**	639	65.3
3. Of the following, which is the correct way to wash hands after handling raw meat?	979	Wipe with towel	2	0.2
		Wash with cold water, wipe dry	14	1.4
		Wash with warm water, wipe dry	25	2.6
		**Wash with soap and warm water, wipe dry**	938	95.8
4. After touching which of the following should you wash your hands during the course of preparing food?	979	Face	10	1
		A pimple on the surface of skin	116	11.8
		Clothes	7	0.7
		**All of the above**	846	86.4
5. People with which of the following symptoms should not cook for others?	979	**Diarrhea, Fever, Sore throat or Flu**	482	49.2
		Skin allergies	56	5.7
		AIDS	435	44.4
		Headache	6	0.6
Pass Rate				83.8%
Mean ± standard deviation				3.6 ± 1.2

**Table 6:** Respondents knowledge on food poisoning, pass rate, mean score, and standard devastation.

Questions	N	Category	Respondents (n.)	Percentage (%)
1. Which of these foodstuffs is more likely to provoke food poisoning if eaten?	979	Fruits taken out of the refrigerator immediately	41	4.2
		Unheated canned food	29	3.0
		**Raw or undercooked beef and eggs**	806	82.3
		Other	103	10.5
2. Which is the most important method for preventing food poisoning?	979	Spray the kitchen with insecticides weekly	29	3
		Avoid eating leftovers	110	11.2
		**Keep food refrigerated until it’s time to serve them**	599	61.2
		Use detergent to disinfect kitchen countertop and stove weekly	241	24.6
3. Can bacteria in food be killed by freezing at −18 °C?	979	Yes, totally	71	7.3
		Yes, partly	235	24
		**Not at all**	184	18.8
		Do not know	489	49.9
4. How to prevent Salmonella poisoning?	979	**Fully heat food**	444	45.4
		Wash food with very hot water	79	8.1
		Freeze food for more than 3 days	57	5.8
		Do not know	399	40.8
5. Which of the following is most likely to become contaminated with *Escherichia Coli* (*E. coli*)?	979	Tap water	28	2.9
		**Raw beef or chicken**	521	53.2
		Raw vegetables	83	8.5
		Do not know	347	35.4
6. Who is more at risk of getting a foodborne illness?	979	Children	119	12.2
		Pregnant women	30	3.1
		Elderly	17	1.7
		**All of the above**	813	83
Pass Rate				78.5%
Mean ± standard deviation				3.5 ± 1.2

**Table 7:** The relationship between the demographic characteristics of participants and their food safety knowledge and practices.

Variables	N	Pass Rate (%)	*p*-Value	Mean Score	*p*-Value
**Age**	979		0.330		0.132
18–25		5%		13.2	
26–35		35.40%		14.1	
36–50		41.60%		14.2	
51 and above		17.90%		14.1	
**How many children do you have?**	979		0.181		0.233
1 child		17.30%		13.7	
2 children		17.60%		14.1	
3 children		19.30%		14.3	
4 children or more		45.70%		14.2	
**Educational level**	979		0.045		0.157
Primary Education		2.10%		13.0	
Secondary Education		21.60%		13.8	
University Degree		76.30%		14.2	
**Employment status**	979		0.891		0.185
Housewife		53.80%		14.3	
Employed		30.20%		13.9	
Retired		9.40%		13.9	
Other		6.60%		14.3	
**What is your monthly income?**	979		0.356		0.158
2000–4999 SR *		11%		13.5	
5000–6999 SR		10.60%		14.0	
7000–9999 SR		21.10%		14.1	
10,000 SR or more		57.30%		14.3	

* SR: Saudi Riyal.
